# Construction Notes

**Published:** 1895-10-05

**Authors:** 


### CONSTRUCTION NOTES.

### New Isolation Hospital at Middlesbrough.

The new block of buildings has cost about £800, and is built on the pavilion system, with two wards and a nurses' room in the centre. A verandah goes the whole length of the structure, which has been fitted with all the latest appliances in the way of heating apparatus, sanitary fittings, &c. The floors are of pitch pine, waxed, the windows being all made in a double form, so as to admit of ventilation without draughts. The new building has been provided for the purpose of isolating cases in which the disease is uncertain, and where it can be diagnosed.

### New "Wilson" Block at the Delancey Hospital, Cheltenham.

The formal opening of the new building at the Delancey Hospital has taken place. The new wards form a wing on the N.E. side of the main building (corresponding with the college wing on the other side), and practically complete the original scheme of the institution, so far as the accommodation for infectious diseases other than diphtheria is concerned. Substantially constructed of brick, like the older buildings, the block is in two storeys, the ground floor being reserved for typhoid and the floor above for additional scarlet fever beds. All the accommodation is for "public" cases—that is, for cases sent in by sanitary authorities. Each floor comprises two principal wards, one for each sex, each of these providing for six adult patients. In addition to these there are two small wards, one on either floor. That on the ground or typhoid floor contains two beds, which will be used for cases requiring special watching. Altogether the equipment of the institution is strengthened to the extent of some thirty beds. The general arrangements of the wing are on the most approved plan. To each of the large wards is attached a room for nurses and attendants, this being fitted with a range for cooking, and a window in the corner commanding a view of the ward. The ground floor is approached by a path, and the wards in the second storey are approached by means of a corridor bridge, from the main hospital. This bridge, together with an external iron staircase which leads up to it, under a covered balcony, serves two purposes: it enables friends to see and speak to the patients, and in the event of fire it is a great safeguard for transit. The ventilation is by means of inlet brackets and extract flues and the warming is by hot-air grates. Including a new stoke-house for heating the hot-water pipes, and supplying the hot-water service to the several blocks, the enlargement has cost nearly £4,000, to which Mr. Charles Wilson (hence the naming of the block), chairman of the trustees, has made the generous contribution of £1,000. The architects are Messrs. Middleton Brothers and Phillott, and the contractors Messrs. Collins and Son, of Tewkesbury.

### New In-patients' Department, Birmingham Ear and Throat Hospital.

An important addition to the work of the Birmingham and Midland Ear and Throat Hospital has been inaugurated lately. In the treatment of ear and throat diseases the need for an in-patient department has all along been strongly felt and the new premises are arranged to meet the demands that such an addition to the work would involve. The committee have opened the in-patient department with ten beds, divided between separate wards for males and females. It will remain to the generous public to provide the means of further extending this all-important department to the thirty beds for which there is available accommodation. In addition to the ordinary wards for in-patients, a quarantine ward is provided high up in the building, with a tube for the removal of infected clothing, and the kitchen and other offices are located high in the building. The fittings of every department are well up to date. The architects are Messrs. J. A. Cousins and Peacock, and the works have been contracted for by Messrs. Gowring and Ingram.

